# Network meta-analysis of curative efficacy of different acupuncture methods on obesity combined with insulin resistance

**DOI:** 10.3389/fendo.2022.968481

**Published:** 2022-09-02

**Authors:** Jiankun Chen, Yingming Gu, Lihong Yin, Minyi He, Na Liu, Yue Lu, Changcai Xie, Jiqiang Li, Yu Chen

**Affiliations:** State Key Laboratory of Dampness Syndrome of Chinese Medicine, The Second Affiliated Hospital of Guangzhou University of Chinese Medicine, Guangdong Provincial Hospital of Chinese Medicine, Guangzhou, China

**Keywords:** acupuncture methods, obesity, insulin resistance, systematic review, network meta-analysis

## Abstract

**Objective:**

This study aims to systematically evaluate the curative efficacy of different acupuncture methods in the treatment of obesity combined with insulin resistance in randomized clinical trials (RCTs) by network meta-analysis.

**Methods:**

Four Chinese databases (CNKI, WanFang Data, VIP, and SinoMed) and four English databases (PubMed, Embase, the Cochrane Library, and www.clinicaltrial.gov) were electronically searched to identify qualified studies. Two reviewers independently screened the literature in accordance with the inclusion/exclusion criteria by EndNote 20 software and extracted data by ADDIS1.16.8 software, and then the risk of bias of the included studies were evaluated by the Cochrane tool. Network meta-analysis was performed by Stata 15.1 software. The primary outcomes included fasting blood glucose (FBG), fasting serum insulin (FINS), homeostasis model assessment—insulin resistance (HOMA-IR), and body mass index (BMI). The secondary outcomes included waistline, waist–hip ratio, triglyceride (TG), total cholesterol (TC), high-density lipoprotein (HDL), and low-density lipoprotein (LDL).

**Results:**

Five RCTs with a total of 410 patients with obesity combined with insulin resistance were included. The results of the network meta-analysis showed that, compared with the control group, three kinds of acupuncture methods (electropuncture, acupoint catgut embedding, and acupuncture point patch) had significant efficacy in reducing FBG [electropuncture (MD = -0.44, 95% CI: -0.83, -0.05) and acupoint catgut embedding (MD = -0.36, 95% CI: -0.51, -0.21)], FINS [electropuncture (MD = -6.17, 95% CI: -9.69, -2.65), acupoint catgut embedding (MD = -5.87, 95% CI: -6.92, -4.82), and acupuncture point patch (MD = -5.86, 95% CI: -11.40, -0.32)], HOMA-IR [electropuncture (MD = -1.59, 95% CI: -2.73, -0.45) and acupoint catgut embedding (MD =-0.91, 95% CI: -1.07, -0.75)], BMI [electropuncture (MD = -1.68, 95% CI: -2.70, -0.66), acupoint catgut embedding (MD = -3.39, 95% CI: -4.38, -2.40), and acupuncture point patch [MD = -2.90, 95%CI: -4.93, -0.87)], and waistline [electropuncture (MD = -5.49, 95% CI: -8.56, -2.42) and acupoint catgut embedding (MD = -4.91, 95% CI: -7.51, -2.31)] in obese patients with insulin resistance, suggesting that their efficacy was better than that of the western medicine group in some of the outcome indicators. For the index related to blood lipid, the efficacy of electropuncture was significantly better than behavioral therapy and western medicine. Except that acupoint catgut embedding was superior to electroacupuncture in reducing the BMI, there was no statistically significant difference in efficacy among the three acupuncture methods.

**Conclusions:**

The results showed that the therapeutic effect of acupuncture methods was superior to conventional western treatment alone. Acupuncture methods could serve as an alternative or adjunctive treatment in obese patients with insulin resistance.

**Systematic Review Registration:**

https://inplasy.com, identifier 202280075.

## Introduction

Obesity, one of the leading health risk factors worldwide, has a prevalence that is rapidly increasing worldwide (1) since 1.1 billion people are classified as overweight (2). Furthermore, obesity is associated with several health problems, including insulin resistance, cardiovascular disease, gallbladder disease, and certain malignancies (3).

Insulin resistance (IR) is the common pathological basis of metabolic diseases such as obesity and type 2 diabetes (4). Obesity and overweight are closely correlated with IR and are independent risk factors for IR (5). The pathogenesis of IR is still unclear, but some studies (6) suggested that it is caused by the interaction between nutritional overload, systemic fatty acid surplus, inflammatory response of adipose tissue, endoplasmic reticulum stress, oxidative stress, and adipose tissue hypoxia.

Acupuncture is the most rapidly growing complementary therapy that is recognized by the WHO (7). In recent years, both experimental and clinical current data concluded that acupuncture was superior to conventional medication for obesity (8) and insulin resistance (9), which can be used to improve symptoms and efficacy while reducing the side effects or adverse reactions caused by western medicine therapy. It is suggested that acupuncture exerts beneficial effects on the mechanisms of obesity and insulin resistance; however, the most effective frequency of obesity combined with insulin resistance by acupuncture remains controversial. Further prospective studies are needed to establish the effectiveness of this complementary method for obesity combined with insulin resistance treatment.

In this study, network meta-analysis was used to systematically evaluate and compare the curative efficacy of different acupuncture methods (electroacupuncture, acupoint catgut embedding, and acupuncture point patch) in the treatment of obesity combined with insulin resistance in randomized clinical trials (RCTs) so as to provide more clinical evidence for the acupuncture treatment of obesity combined with insulin resistance and to guide clinicians in sophisticated treatment options.

## Data and methods

### Criteria for considering studies for this study

#### Type of studies

RCTs of different acupuncture methods in the treatment of obesity with insulin resistance, blind method, and language are not limited.

#### Type of participants

The patients were diagnosed to be obese with insulin resistance. The obesity references, the Consensus of Experts on the Prevention and Treatment of Adult Obesity in China in 2011 and the Consensus of Chinese Experts on Medical Nutrition Therapy for Overweight/Obesity in 2016, were developed by the Obesity Group of the Chinese Society of Endocrinology (BMI ≥ 28). For the IR reference, according to the Expert Opinions on Insulin Resistance Evaluation published by the Chinese Diabetes Society, HOMA-IR ≥2.68 is regarded as the standard for the diagnosis of IR, regardless of age, gender, and course of disease.

#### Type of interventions

##### Control group

In terms of other acupuncture treatments, drug therapy, or blank control, the experimental group consisted of those who have had any kind of acupuncture, moxibustion, acupuncture + moxibustion, warm acupuncture, electropuncture, auricular point, acupoint application, and acupoint catgut embedding. In addition to intervention measurements, other background treatment measurements were identical in both groups.

#### Type of outcome measures

##### Primary outcomes

These included (1) fasting blood glucose (FBG), (2) fasting serum insulin (FINS), (3) homeostasis model assessment—IR (HOMA-IR), and (4) body mass index (BMI).

##### Secondary outcomes

These included (1) waistline, (2) waist–hip ratio, (3) triglyceride (TG), (3) total cholesterol (TC), (5) high-density lipoprotein (HDL), and (6) low-density lipoprotein (LDL).

#### Exclusion criteria

These included (1) non-RCT research: descriptive studies, case–control studies, cohort studies, literature review, social commentary, case reports, case series analysis, *etc.*; (2) intervention measures that take a variety of therapy combination or study acupuncture and moxibustion different points, different techniques, the study of frequency; (3) animal studies, cellular or analytical studies, or systematic reviews, meta-analyses, and pooled analyses of multiple RCTs; (4) subjects who suffered from serious diseases, such as cerebrovascular diseases and tumors; and (5) others: conference abstracts, comments, guidelines, letters, amendments, and other unrelated studies where the full text is not available, and the results are incomplete.

### Literature retrieval

Cross-retrieval of the Chinese databases (CNKI, WanFang Data, VIP, and SinoMed) and the English databases (PubMed, Embase, the Cochrane Library, and www.clinicaltrial.gov) was performed by electronically searching from database construction time to March 31, 2022.

The keywords or mesh terms used were as follows: acupuncture, needle, electroacupuncture, moxibustion, fire needle, needle warming moxibustion, auricular point, point application therapy, acupoint catgut embedding, obesity, insulin resistance, controlled clinical trial, randomized controlled trial, drug therapy, groups, and placebo. Considering that there may be differences in the description of outcomes in the RCT, outcome indicators were not restricted in the retrieval to avoid omission.

### Literature management

By aggregating the studies retrieved from various archives, we used EndNote 20 software to manage the retrieved literature. After excluding the literatures duplicated between different databases, two researchers independently read the title and abstract of the literatures, screened out the obvious irrelevant literatures according to the inclusion/exclusion criteria, and screened the literatures by reading the full text if necessary. The screening results are cross-checked by two researchers, and in case of disagreement, consultation or discussion is done with a third expert.

### Literature quality evaluation and data extraction

We used Excel 2016 software to develop basic information extraction table and quality evaluation table. Two reviewers independently conducted quality evaluation and basic data extraction for each article that met the inclusion criteria. Detailed data, including basic study information (author, publication year, study type, sample size, *etc.*), intervention measures and outcome indicators, quality evaluation, *etc.*, were extracted by ADDIS1.16.8 software. Two reviewers cross-checked the results, and if there is any disagreement, it shall be decided through a discussion or consultation with a third reviewer.

### Bias risk assessment of included studies

Two reviewers assessed the risk of bias in the included studies according to the Cochrane Manual’s risk of bias assessment tool for RCTs. The projects include randomization of assignment methods, assignment plan concealment, blinding of study subjects and protocol implementors, blinding of study outcome measures, integrity of outcome data, selective reporting of study results, and other sources of bias. Finally, the risk of literature bias was judged as “low”, “high”, and “uncertain”. Two reviewers independently conducted the assessment, and then this was cross-checked, in case of disagreement, through a discussion with the third reviewer to discuss decisions, thus reaching a consensus.

### Statistical analysis

Using Stata 15.1 software and its “network” commands to draw the network diagram for comparison between intervention measures for the evaluation of publication bias, a network meta-analysis was conducted for each outcome, and heterogeneity and inconsistency in the mesh evidence body were tested. In this study, odds ratio (OR) and 95% confidence interval (CI) were used as a way of expression for the dichotomous outcome index, while the continuous outcome index was expressed as mean difference (MD) and its 95% confidence interval.

The existence of a publication bias was identified by drawing a corrected comparison funnel plot, and the inconsistencies of the results of the mesh meta-analysis were tested by a node splitting method. If the direct comparison and the indirect comparison result in a difference of *P* > 0.05, the inconsistency is not significant, and the consistency model is adopted. At the same time, a prediction interval graph was drawn for each outcome. If the prediction interval crossed the invalid line, interstudy heterogeneity was considered, and the random effect model was selected. Efficacy ranking was based on the Surface under the Cumulative Ranking Curve (SUCRA). The larger the SUCRA is, the better the efficacy of the drug in this outcome. *P <*0.05 was considered statistically significant.

## Results

### Literature screening process and results

**Figure 1:**
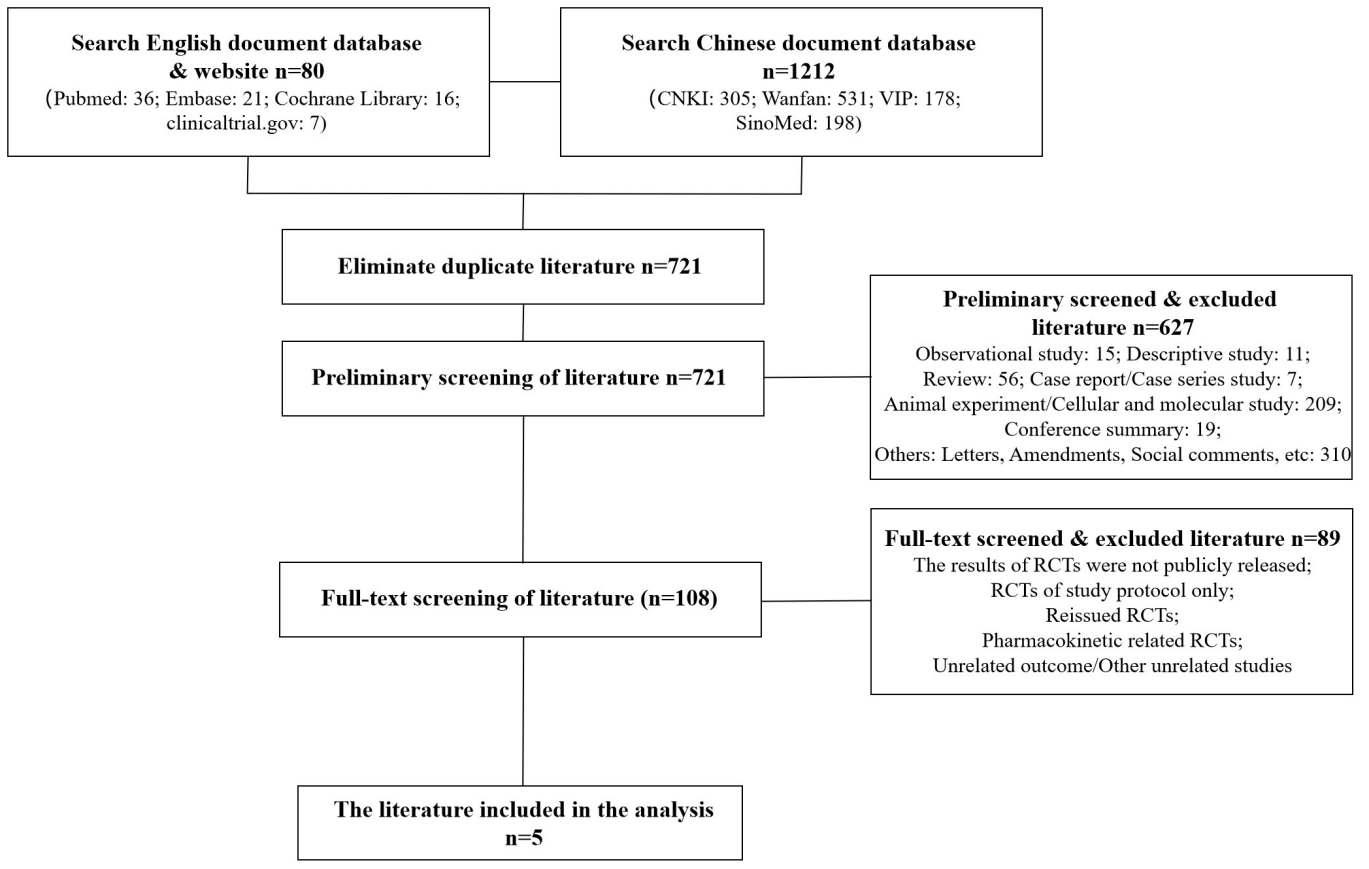
Literature screening process and results.

A total of 627 related articles were initially detected, and 89 literatures were detected after full-text screening. Finally, 5 RCTs (10–14) were included in the network meta-analysis, including 410 patients on obesity combined with insulin resistance. The specific literature screening flow chart and results are shown in **Figure 1**.

### Basic characteristics of included studies

The basic characteristics of the included studies are detailed in the Open Science (Resource Services) Identifier (OSID). Five RCTs included three types of intervention (electropuncture, acupoint catgut embedding, and acupuncture point patch) and control groups (behavioral therapy, western medicine, and blank control). The mean age of the 410 patients included in this study was 33.04 ± 7.45 years, and the mean duration of disease was 4.92 ± 3.20 years. The basic characteristics of the included studies are shown in **Supplementary Table S1**. As shown in the table, outcomes available for network meta-analysis included FBG, FINS, HOMA-IR, BMI, waistline, waist–hip ratio, TG, TC, HDL, and LDL.

### Results of network meta-analysis

#### Fasting blood glucose

**Figure 2:**
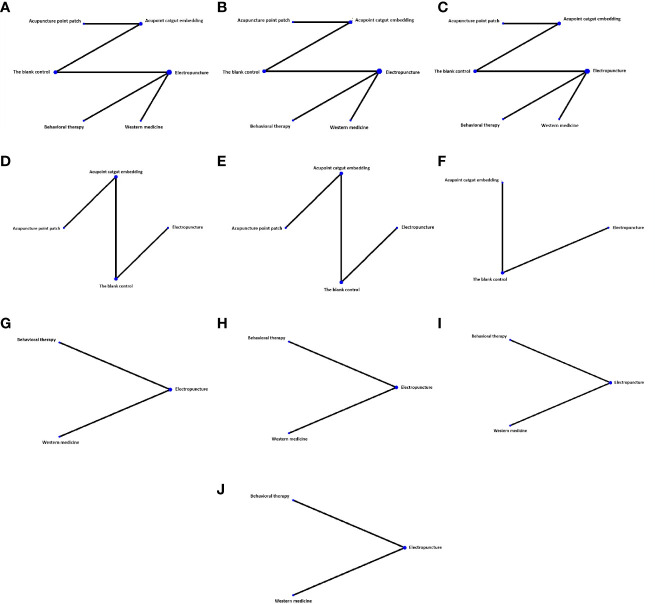
Network diagram of different indexes treated by different types of intervention. The figure shows that each intervention is indicated with a single blue dot. The size of the dot represents the cumulative total sample size of the intervention. The line segment between the dots represents the studies that have a direct comparison between the two interventions. **(A)** Fasting blood glucose, **(B)** fasting serum insulin, **(C)** homeostasis model assessment—insulin resistance, **(D)** BMI, **(E)** waistline, **(F)** waist–hip ratio, **(G)** triglyceride, **(H)** total cholesterol, **(I)** high-density lipoprotein, and **(J)** low-density lipoprotein.

**Figure 3:**
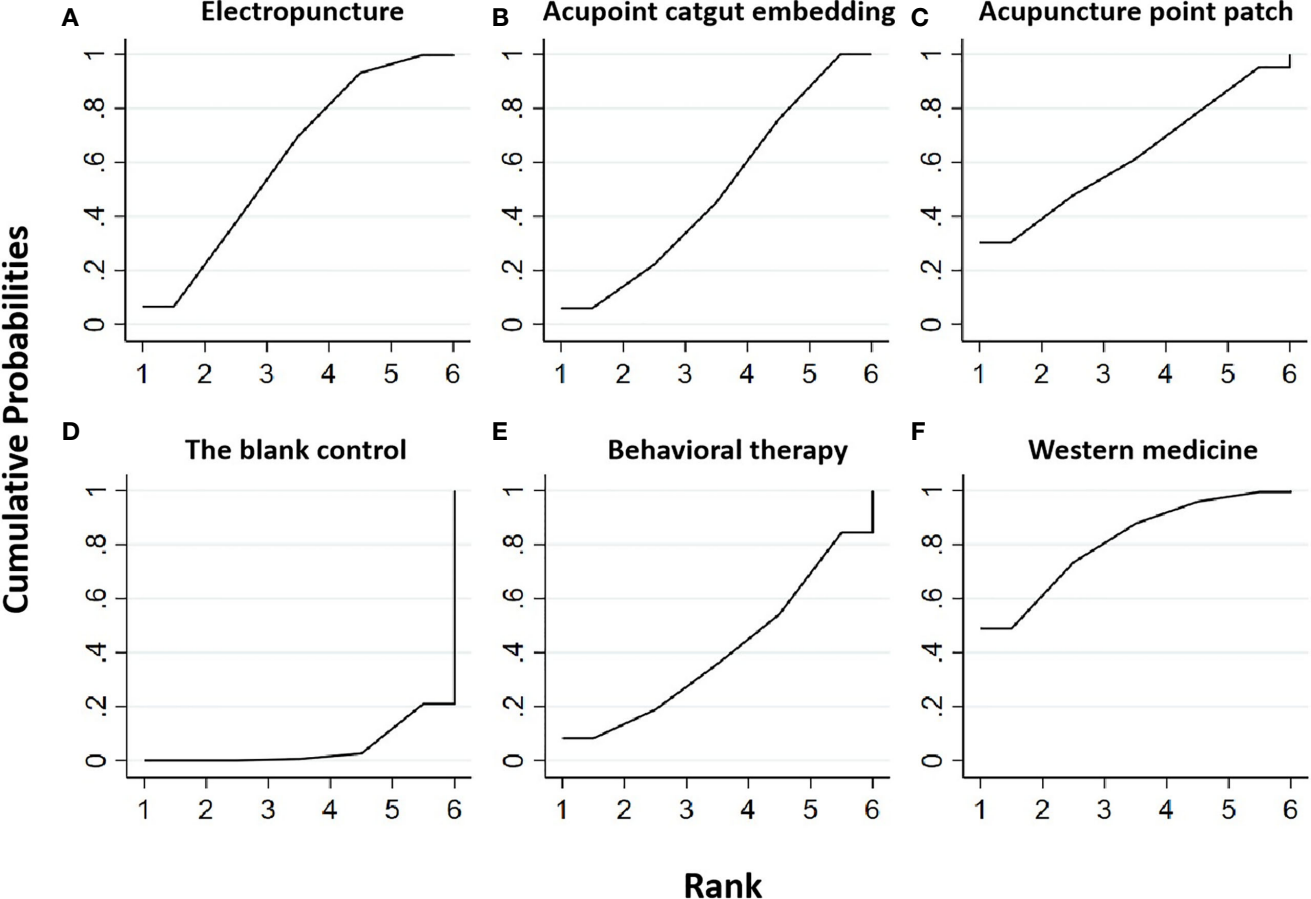
Efficacy ranking and cumulative probability graph of fasting blood glucose (FBG) treated by different types of intervention. Comparison of efficacy ranking and cumulative probability of FBG between each kind of intervention. **(A)** Electropuncture, **(B)** acupoint catgut embedding, **(C)** acupuncture point patch, **(D)** the blank control, **(E)** behavioral therapy, and **(F)** western medicine.

Five RCTs were included in the statistical analysis of FBG, with a total sample of 410 patients. The network relationship is shown in **Figure 2A**; the thickness of the line segment represents the number of studies included in the comparison of each treatment method, and the circular area represents the sample size of the population using this measure. The line segments between the dots represent studies in which there was a direct comparison between the two interventions that were connected. No significant heterogeneity or inconsistency was found in the reticular body of evidence; thus we adopted the consistent fixed-effect model. The pairwise comparison results and ranking of reticular meta-analysis are shown in **Figure 3** and **Tables 1**, **2**.

**Table 2:** Network meta-analysis netleague table of fasting blood glucose.

**Electropuncture**	0.08 (-0.34, 0.50)	-0.02 (-0.69, 0.65)	0.14 (-0.28, 0.56)	-0.14 (-0.47, 0.19)	0.44 (0.05, 0.83)
-0.08 (10.50, 0.34)	**Acupoint catgut embedding**	-0.10 (-0.63, 0.43)	0.06 (-0.53, 0.66)	-0.22 (-0.76, 0.32)	0.36 (0.21, 0.51)
0.02 (-0.65, 0.69)	0.10 (-0.43, 0.63)	**Acupuncture point patch**	0.16 (-0.63, 0.95)	-0.12 (-087, 0.63)	0.46 (-0.09, 1.01)
-0.14 (-0.56,0.28)	-0.06 (-0.66, 0.53)	-0.16 (-0.95, 0.63)	**Behavioral therapy**	-0.28 (-0.82, 0.26)	0.30 (-0.27, 0.87)
0.14 (-0.19, 0.47)	0.22 (-0.32, 0.76)	0.12 (-0.63, 0.87)	0.28 (-0.26, 0.82)	**Western medicine**	0.58 (0.07, 1.09)
-0.44 (-0.83, -0.05)	-0.36 (-0.51, -0.21)	-0.46 (-1.01, 0.09)	-0.30 (-0.87, 0.27)	-0.58 (-1.09, -0.07)	**The blank control**

Bold values means the sorting tables represent the area under the curve of the cumulative probability sorting chart, the bold values are the rank of the sorting by different types of intervention in our study. While the number of the ladder table is the relative effect value and 95% CI.

**Table 1:** Efficacy ranking of mesh meta-analysis: fasting blood glucose and fasting serum insulin.

Treatment	SUCRA	Rank
Fasting blood glucose		
Western medicine	81.1	1
Acupuncture point patch	62.5	2
Electropuncture	61.3	3
Acupoint catgut embedding	49.8	4
Behavioral therapy	40.3	5
Blank control	4.9	6
Fasting serum insulin		
Behavioral therapy	83.2	1
Electropuncture	65.8	2
Acupuncture point patch	65.5	3
Acupoint catgut embedding	64.4	4
Blank control	13.2	5
Western medicine	7.9	6

As shown in the table, the surface under the cumulative ranking curve (SUCRA) represents the area under the curve of the cumulative probability graph of efficacy ranking. The decrease in fasting blood glucose was better, the SUCRA value was bigger, and the efficacy ranking was higher.

**Table 3** is about the pairwise comparison results of six types of interventions or controls, which showed that, compared with the blank control, electropuncture (MD = -0.44, 95% CI: -0.83, -0.05), acupoint catgut embedding (MD = -0.36, 95% CI: -0.51, -0.21), and western medicine (MD = -0.58, 95% CI: -1.09, -0.07) groups all had reduced FBG, while no statistical differences were found in the other groups. From **Figure 3** and **Table 1**, we found that western medicine ranked first in terms of reducing FBG in obesity combined with insulin resistance, while acupuncture point patch, electropuncture, and acupoint catgut embedding ranked second, third, and fourth, respectively.

**Table 3:** Network meta-analysis ladder table of fasting serum insulin.

**Electropuncture**	0.30 (-3.37, 3.97)	0.31 (-6.26, 6.87)	-0.78 (-2.38, 0.82)	6.87 (5.65, 8.09)	6.17 (2.65, 9.69)
-0.3 (-3.97, 3.37)	**Acupoint catgut embedding**	0.01 (-2.93, 5.08)	-1.08 (-5.08, 2.93)	6.57 (2.70, 10.44)	5.87 (4.82, 6.92)
-0.31 (-6.87, 6.26)	-0.01 (-5.45, 5.43)	**Acupuncture point patch**	-1.09 (-7.84, 5.67)	6.56 (-0.12, 13.24)	5.86 (0.32, 11.40)
0.78 (-0.82, 2.38)	1.08 (-2.93, 5.08)	1.09 (-5.67, 7.84)	**Behavioral therapy**	7.65 (5.64, 9.66)	6.95 (3.08, 10.82)
-6.87 (-8.09, -5.65)	-6.57 (-10.44, -2.70)	-6.56 (-13.24, 0.12)	-7.65 (-9.66, -5.64)	**Western medicine**	-0.70 (-4.43, 3.02)
-6.17 (-9.69, -2.65)	-5.87 (-6.92, -4.82)	-5.86 (-11.40, -0.32)	-6.95 (-10.82, -3.08)	0.70 (-3.02, 4.43)	**The blank control**

Bold values means the sorting tables represent the area under the curve of the cumulative probability sorting chart, the bold values are the rank of the sorting by different types of intervention in our study. While the number of the ladder table is the relative effect value and 95% CI.

#### Fasting serum insulin

**Figure 4:**
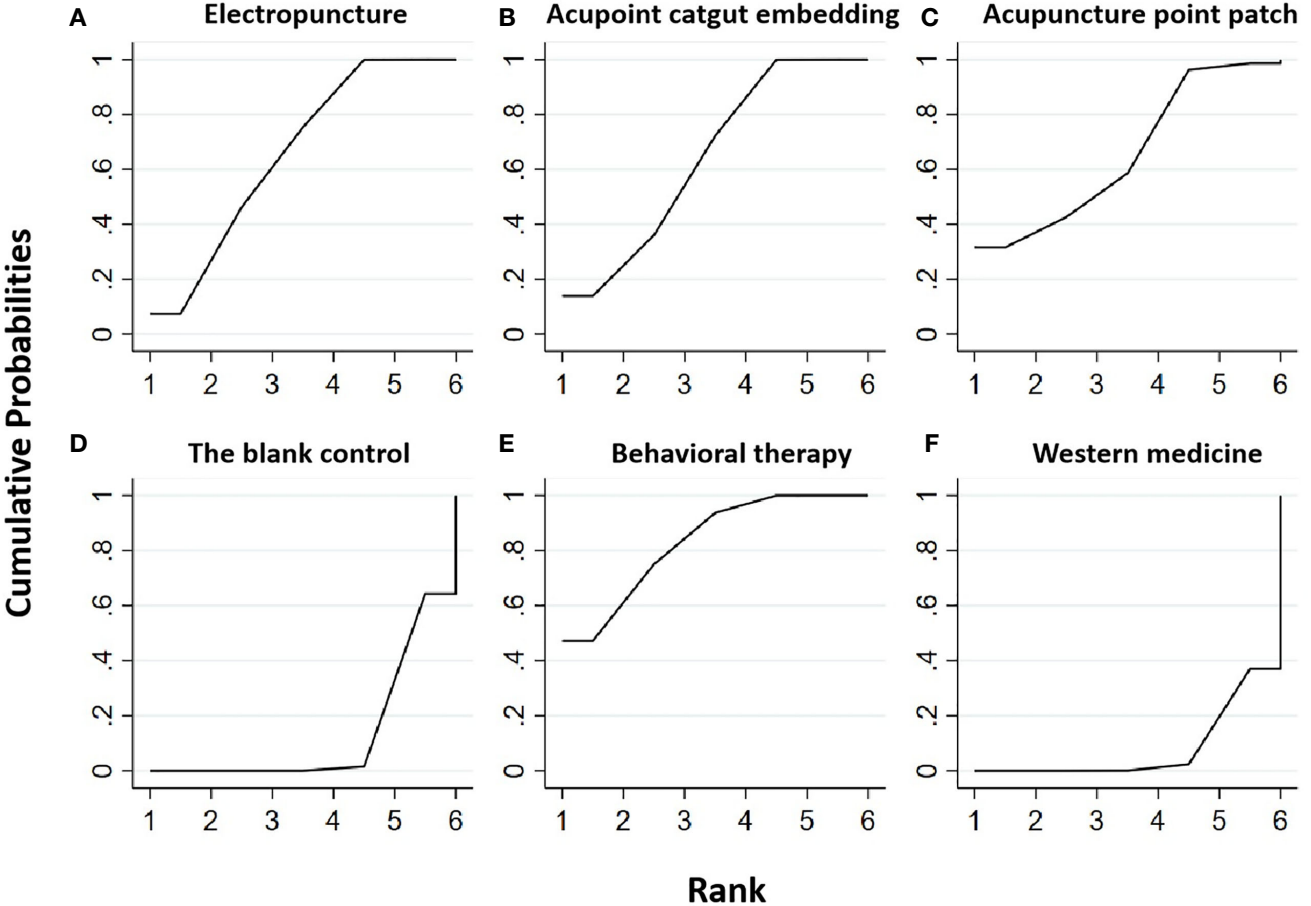
Efficacy ranking and cumulative probability graph of fasting serum insulin (FINS) treated by different types of intervention. Comparison of efficacy ranking and cumulative probability of FINS between each kind of intervention. **(A)** Electropuncture, **(B)** acupoint catgut embedding, **(C)** acupuncture point patch, **(D)** blank control, **(E)** behavioral therapy, and **(F)** western medicine.

Five RCTs were included in the statistical analysis of FINS, with a total sample of 410 patients. The network relationship is shown in **Figure 2B**; no significant heterogeneity or inconsistency was found in the reticular body of evidence; thus, the consistent fixed effect model was adopted. The pairwise comparison results and ranking of reticular meta-analysis are shown in **Figure 4** and **Tables 1**, **2**.

**Table 2** shows that, compared with the blank control, the electropuncture (MD = -6.17, 95% CI: -9.69, -2.65), acupoint catgut embedding (MD = -5.87, 95% CI: -6.92, -4.82), acupuncture point patch (MD =-5.86, 95% CI: -11.40, -0.32), and behavioral therapy (MD = -6.95, 95%CI: -10.82, -3.08) groups all caused the decrease in the FINS level. However, compared with western medicine, the electropuncture (MD = -6.87, 95% CI: -8.09, -5.65), acupoint catgut embedding (MD = -6.57, 95% CI: -10.44, -2.70), and behavioral therapy (MD = -7.65, 95% CI: -9.66, -5.64) groups all had better curative efficacy. No statistical differences were found in the other groups. From **Figure 4** and **Table 1**, we found that behavioral therapy ranked first in terms of reducing FINS in obesity combined with insulin resistance, while electropuncture, acupuncture point patch, and acupoint catgut embedding ranked second, third, and fourth, respectively.

#### Homeostasis model assessment-IR

**Figure 5:**
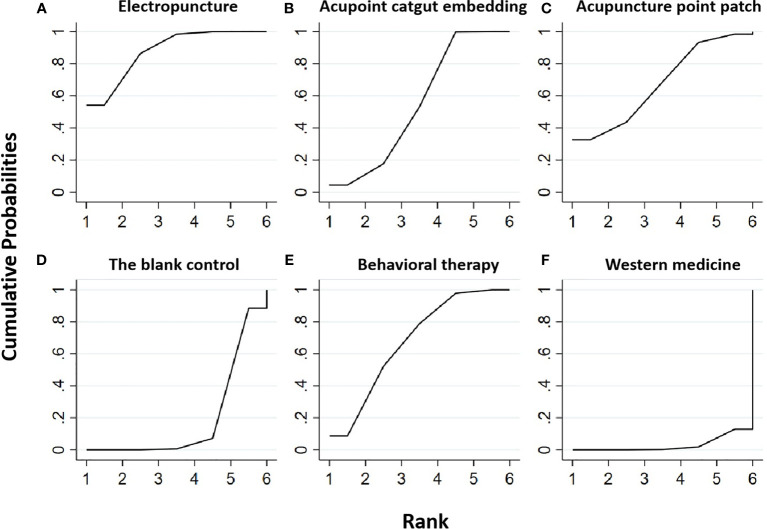
Efficacy ranking and cumulative probability graph of homeostasis model assessment—insulin resistance (HOMA-IR) treated by different types of intervention. Comparison of efficacy ranking and cumulative probability of HOMA-IR between each kind of intervention. **(A)** Electropuncture, **(B)** acupoint catgut embedding, **(C)** acupuncture point patch, **(D)** blank control, **(E)** behavioral therapy, and **(F)** western medicine.

Five RCTs were included in the statistical analysis of HOMA-IR, with a total sample of 410 patients. The network relationship is shown in **Figure 2C**. No significant heterogeneity or inconsistency was found in the reticular body of evidence; thus, the consistent fixed-effect model was adopted. The pairwise comparison results and ranking of reticular meta-analysis are shown in **Figure 5** and **Tables 4**, **5**.

**Table 4:** Efficacy ranking of mesh meta-analysis: homeostasis model assessment—insulin resistance (HOMA-IR) and body mass index (BMI).

Treatment	SUCRA	Rank
HOMA-IR		
Electropuncture	87.8	1
Behavioral therapy	67.5	2
Acupuncture point patch	67.4	3
Acupoint catgut embedding	55	4
Blank control	19.3	5
Western medicine	3	6
BMI		
Acupoint catgut embedding	89.9	1
Acupuncture point patch	71.5	2
Electropuncture	38.5	3
Blank control	0.1	4

As shown in the table, the surface under the cumulative ranking curve (SUCRA) represents the area under the curve of the cumulative probability graph of efficacy ranking. The decrease in fasting blood glucose is better, the SUCRA value is bigger, and the efficacy ranking is higher.

**Table 5:** Network meta-analysis ladder table of homeostasis model assessment—insulin resistance.

**Electropuncture**	0.68 (-0.47, 1.83)	0.38 (-1.50, 2.25)	0.29 (-0.22, 0.80)	2.30 (1.92, 2.68)	1.59 (0.45, 2.73)
-0.68 (-1.83, 0.47)	**Acupoint catgut embedding**	-0.30 (-1.78, 1.18)	-0.39 (-1.64, 0.87)	1.62 (0.41, 2.83)	0.91 (0.75, 1.07)
-0.38 (-2.25, 1.50)	0.30 (-1.18, 1.78)	**Acupuncture point patch**	-0.09 (-2.03, 1.85)	1.92 (0.01, 3.83)	1.21 (-0.28, 2.70)
-0.29 (-0.80. 0.22)	0.39 (-0.87, 1.64)	0.09 (-1.85, 2.03)	**Behavioral therapy**	2.01 (1.37, 2.65)	1.30 (0.05, 2.54)
-2.30 (-2.68, -1.92)	-1.62 (-2.83, -0.41)	-1.92 (-3.83, -0.01)	-2.01 (-2.65, -1.37)	**Western medicine**	-0.71 (-1.91, 0.49)
-1.59 (-2.73, -0.45)	-0.91 (-1.07, -0.75)	-1.21 (-2.70, 0.28)	-1.30 (-2.54, -0.05)	0.71 (-0.49, 1.91)	**The blank control**

Bold values means the sorting tables represent the area under the curve of the cumulative probability sorting chart, the bold values are the rank of the sorting by different types of intervention in our study. While the number of the ladder table is the relative effect value and 95% CI.

**Table 5** shows that, compared with the blank control, the electropuncture (MD = -1.59, 95% CI: -2.73, -0.45), acupoint catgut embedding (MD = -0.91, 95% CI: -1.07, -0.75), and behavioral therapy (MD = -1.30, 95% CI: -2.54, -0.05) groups all caused the decrease in the HOMA-IR level. However, compared with western medicine, the electropuncture (MD = -2.30, 95% CI: -2.68, -1.92), acupoint catgut embedding (MD = -1.62, 95% CI: -2.83, -0.41), acupuncture point patch (MD = -1.92, 95% CI: -3.83, -0.01), and behavioral therapy (MD = -2.01, 95% CI: -2.65, -1.37) groups all had better curative efficacy. No statistical differences were found in the other groups. From **Figure 5** and **Table 4**, we found that electropuncture ranked first in terms of reducing HOMA-IR in obesity combined with insulin resistance, while behavioral therapy, acupuncture point patch, and acupoint catgut embedding ranked second, third, and fourth, respectively.

#### Body mass index

**Figure 6:**
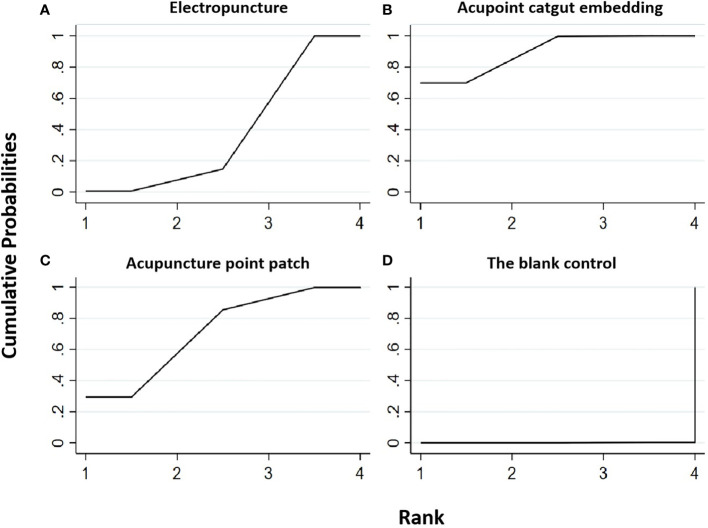
Efficacy ranking and cumulative probability graph of body mass index (BMI) treated by different types of intervention. Comparison of efficacy ranking and cumulative probability of BMI between each kind of intervention. **(A)** Electropuncture, **(B)** acupoint catgut embedding, **(C)** acupuncture point patch, and **(D)** blank control.

Three RCTs were included in the statistical analysis of BMI, with a total sample of 247 patients. The network relationship is shown in **Figure 2D**. No significant heterogeneity or inconsistency was found in the reticular body of evidence; thus, the consistent fixed-effect model was adopted. The pairwise comparison results and ranking of reticular meta-analysis are shown in **Figure 6** and **Tables 4**, **6**.

**Table 6:** Network meta-analysis ladder table of body mass index.

**Electropuncture**	-1.71 (-3.13, 0.29)	-1.22 (-3.49, 1.05)	1.68 (0.66, 2.70)
1.71 (0.29,3.13)	**Acupoint catgut embedding**	0.49 (-1.29, 2.27)	3.39 (2.40, 4.38)
1.22 (-1.05, 3.49)	-0.49 (-2.27, 1.29)	**Acupuncture point patch**	2.90 (0.87, 4.93)
-1.68 (-2.70, -0.66)	-3.39 (-4.38, -2.40)	-2.90 (-4.93, -0.87)	**The blank control**

Bold values means the sorting tables represent the area under the curve of the cumulative probability sorting chart, the bold values are the rank of the sorting by different types of intervention in our study. While the number of the ladder table is the relative effect value and 95% CI.

**Table 6** shows that, compared with blank control, the electropuncture (MD = -1.68, 95% CI: -2.70, -0.66), acupoint catgut embedding (MD = -3.39, 95% CI: -4.38, -2.40), and acupuncture point patch (MD = -2.90, 95% CI (-4.93, -0.87) groups all caused the decrease in the BMI level. However, the curative efficacy of electropuncture (MD = 1.71, 95% CI: 0.29, 3.13) was not as good as acupoint catgut embedding. No statistical differences were found in the other groups. From **Figure 6** and **Table 4**, we found that acupoint catgut embedding ranked first in terms of reducing BMI in obesity combined with insulin resistance, while acupuncture point patch and electropuncture ranked second and third, respectively.

#### Waistline

**Figure 7:**
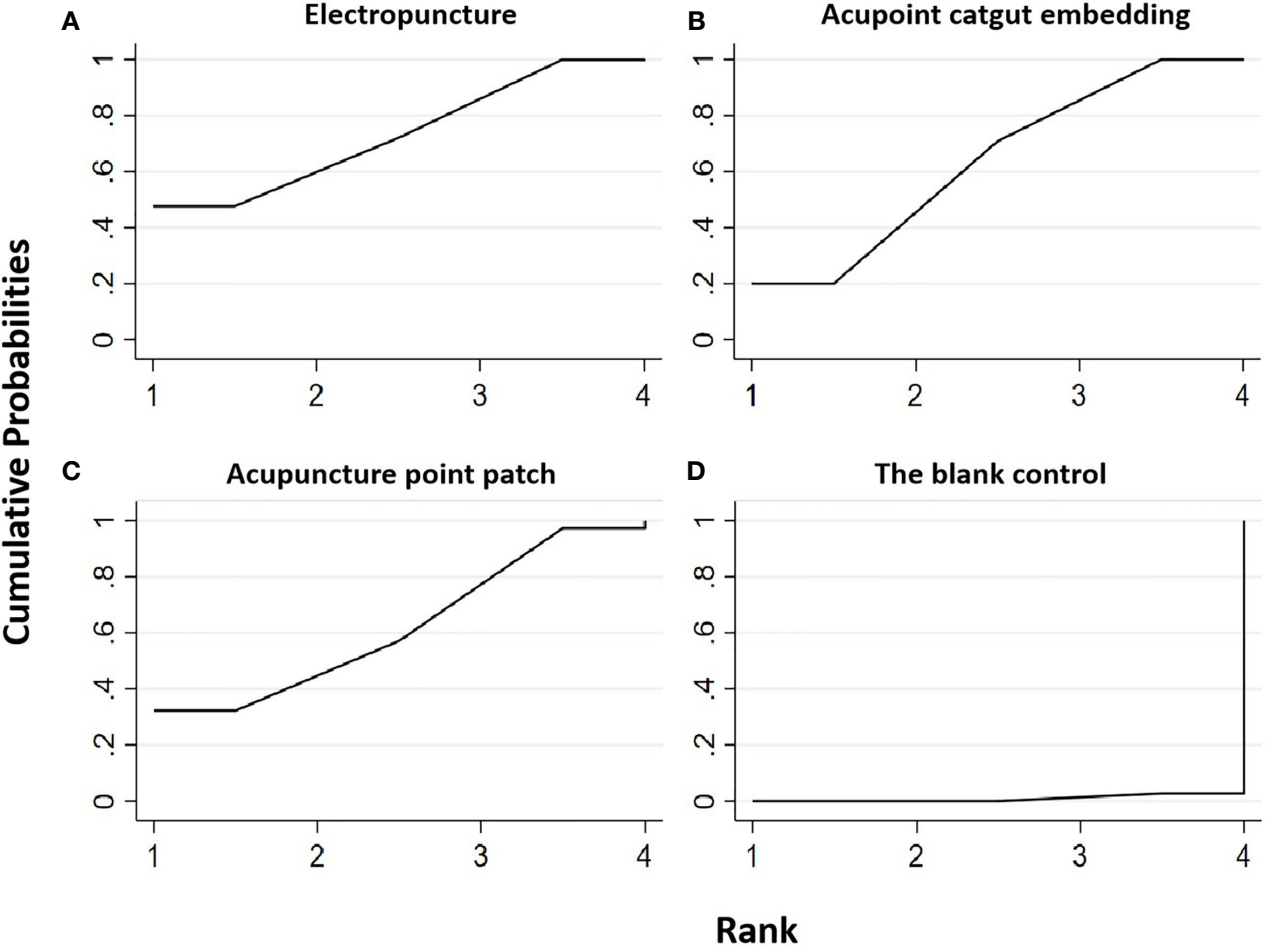
Efficacy ranking and cumulative probability graph of waistline treated by different types of intervention. Comparison of efficacy ranking and cumulative probability of waistline between each kind of intervention. **(A)** Electropuncture, **(B)** acupoint catgut embedding, **(C)** acupuncture point patch, and **(D)** blank control.

Three RCTs were included in the statistical analysis of waistline, with a total sample of 247 patients. The network relationship is shown in **Figure 2E**. No significant heterogeneity or inconsistency was found in the reticular body of evidence; thus, the consistent fixed-effect model was adopted. The pairwise comparison results and ranking of reticular meta-analysis are shown in **Figure 7** and **Tables 7**, **8**.

**Table 7:** Efficacy ranking of mesh meta-analysis: waistline and waist–hip ratio.

Treatment	SUCRA	Rank
Waistline		
Electropuncture	73.2	1
Acupoint catgut embedding	63.6	2
Acupuncture point patch	62.3	3
Blank control	0.9	4
Waist-hip ratio		
Acupoint catgut embedding	100	1
Electropuncture	50	2
Blank control	0	3

As shown in the table, the surface under the cumulative ranking curve (SUCRA) represents the area under the curve of the cumulative probability graph of efficacy ranking. The decrease in fasting blood glucose is better, the SUCRA value is bigger, and the efficacy ranking is higher.

**Table 8:** Network meta-analysis ladder table of waistline.

Electropuncture	0.58 (-3.44, 4.60)	0.72 (-5.06, 6.50)	5.49 (2.42, 8.56)
-0.58 (-4.60, 3.44)	Acupoint catgut embedding	0.14 (-4.01, 4.29)	4.91 (2.31, 7.51)
-0.72 (-6.50, 5.06)	-0.14 (-4.29, 4.01)	Acupuncture point patch	4.77 (-0.13, 9.67)
**-5.49 (-8.56, -2.42)**	**-4.91 (-7.51, -2.31)**	-4.77 (-9.67, 0.13)	Blank control

Bold values means the sorting tables represent the area under the curve of the cumulative probability sorting chart, the bold values are the rank of the sorting by different types of intervention in our study. While the number of the ladder table is the relative effect value and 95% CI.

**Table 8** shows that, compared with blank control, electropuncture (MD = -5.49, 95% CI: -8.56, -2.42) and acupoint catgut embedding (MD = -4.91, 95% CI: -7.51, -2.31) both caused the decrease in the waistline level. No statistical differences were found in the other groups. From **Figure 7** and **Table 7**, we found that electropuncture ranked first in terms of reducing waistline in obesity combined with insulin resistance, while acupoint catgut embedding and acupuncture point patch ranked second and third, respectively.

#### Waist–hip ratio

**Figure 8:**
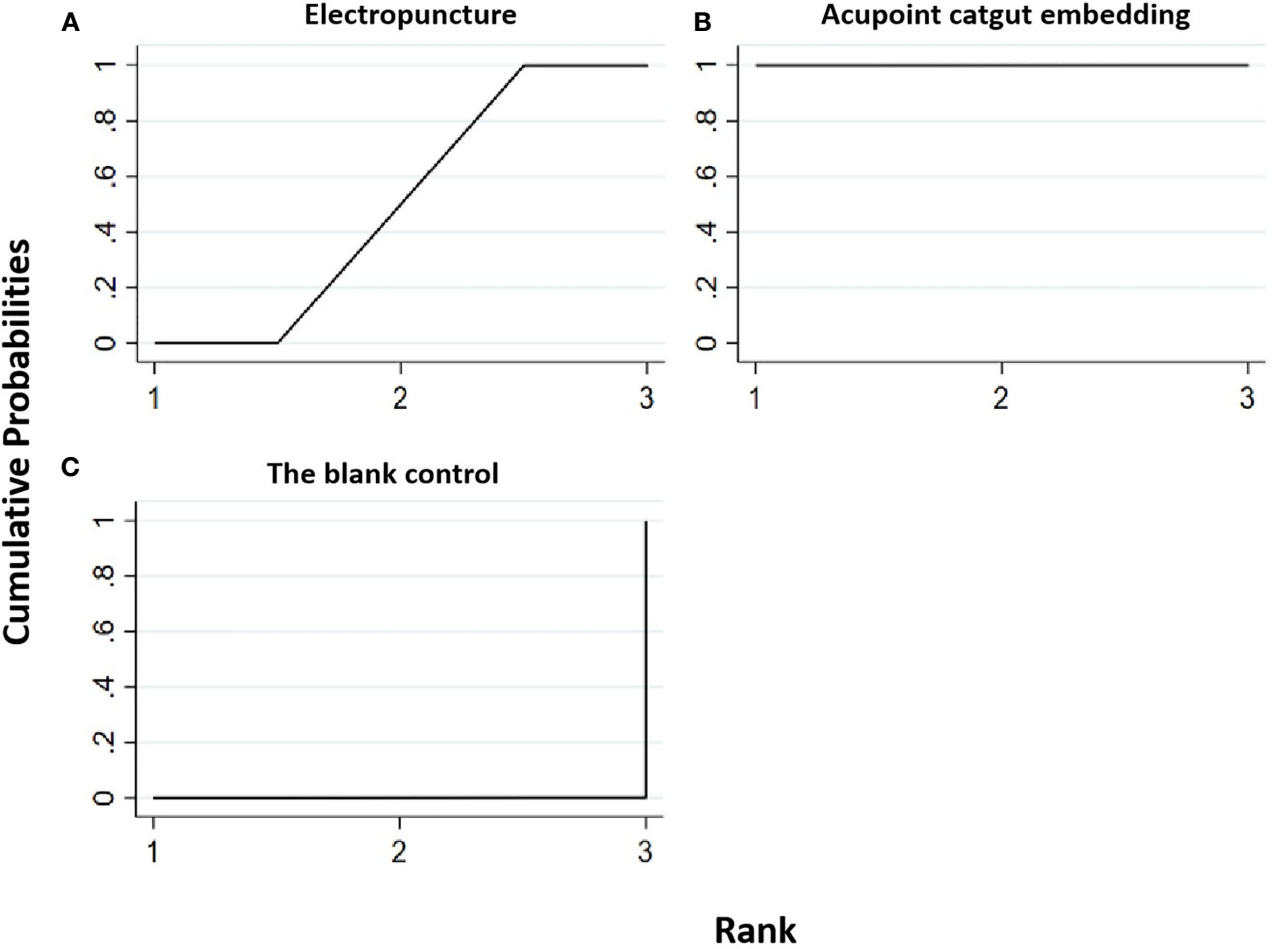
Efficacy ranking and cumulative probability graph of waist–hip ratio treated by different types of intervention. Comparison of efficacy ranking and cumulative probability of waist–hip ratio between each kind of intervention. **(A)** Electropuncture, **(B)** acupoint catgut embedding, and **(C)** blank control.

Two RCTs were included in the statistical analysis of the waist–hip ratio, with a total sample of 185 patients. The network relationship is shown in **Figure 2F**. No significant heterogeneity or inconsistency was found in the reticular body of evidence; thus, the consistent fixed-effect model was adopted. The pairwise comparison results and ranking of reticular meta-analysis are shown in **Figure 8** and **Tables 7**, **9**.

**Table 9:** Network meta-analysis ladder table of waist–hip ratio.

Electropuncture	-0.06 (-0.09, -0.03)	0.02 (0.01, 0.03)
0.06 (0.03, 0.09)	Acupoint catgut embedding	0.08 (0.05, 0.11)
-0.02 (-0.03, -0.01)	-0.08 (-0.11, -0.05)	Blank control

**Table 9** shows that, compared with blank control, electropuncture (MD = -0.02, 95% CI: -0.03, -0.01) and acupoint catgut embedding (MD = -0.08, 95% CI: -0.11, -0.05) both caused the decrease in the waist–hip ratio level. No statistical differences were found in the other groups. From **Figure 8** and **Table 7**, we found that acupoint catgut embedding ranked first in terms of reducing the waist–hip ratio in obesity combined with insulin resistance, while electropuncture ranked second.

#### Triglyceride

**Figure 9:**
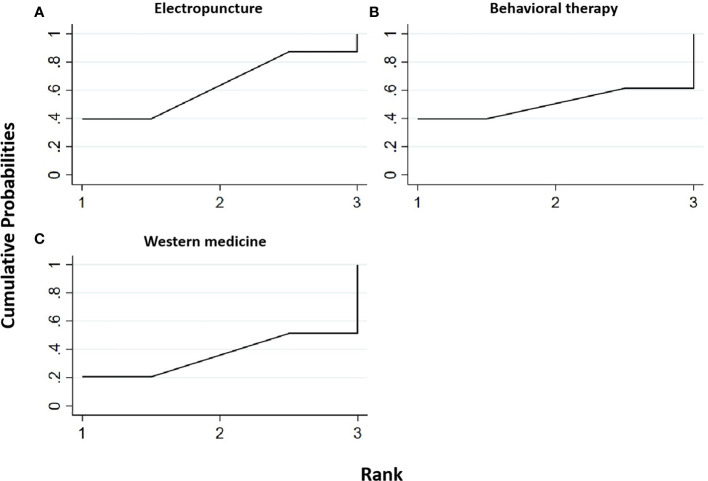
Efficacy ranking and cumulative probability graph of triglyceride (TG) treated by different types of intervention. Comparison of efficacy ranking and cumulative probability of TG between each kind of intervention. **(A)** Electropuncture, **(B)** behavioral therapy, and **(C)** western medicine.

Two RCTs were included in the statistical analysis of TG, with a total sample of 163 patients. The network relationship is shown in **Figure 2G**. No significant heterogeneity or inconsistency was found in the reticular body of evidence; thus, the consistent fixed-effect model was adopted. The pairwise comparison results and ranking of reticular meta-analysis are shown in **Figure 9** and **Tables 10**, **11**.

**Table 10:** Efficacy ranking of mesh meta-analysis: triglyceride and total cholesterol.

Treatment	SUCRA	Rank
Triglyceride		
Electropuncture	63.5	1
Behavioral therapy	50.6	2
Western medicine	36	3
Total cholesterol		
Electropuncture	74.3	1
Western medicine	73.4	2
Behavioral therapy	2.3	3

As shown in the table, surface under the cumulative ranking curve (SUCRA) represents the area under the curve of the cumulative probability graph of efficacy ranking. The decrease in fasting blood glucose is better, the SUCRA value is bigger, and the efficacy ranking is higher.

**Table 11:** Network meta-analysis ladder table of triglyceride.

Electropuncture	0.02 (-0.21, 0.25)	0.04 (-0.11, 0.19)
-0.02 (-0.25, 0.21)	Behavioral therapy	0.02 (-0.25, 0.29)
-0.04 (-0.19, 0.11)	-0.02 (-0.29, 0.25)	Western medicine

**Table 11** shows that, compared with blank control, electropuncture, behavioral therapy, and western medicine all had no statistical differences. From **Figure 9** and **Table 10**, we found that electropuncture ranked first in terms of reducing TG in obesity combined with insulin resistance.

#### Total cholesterol

**Figure 10:**
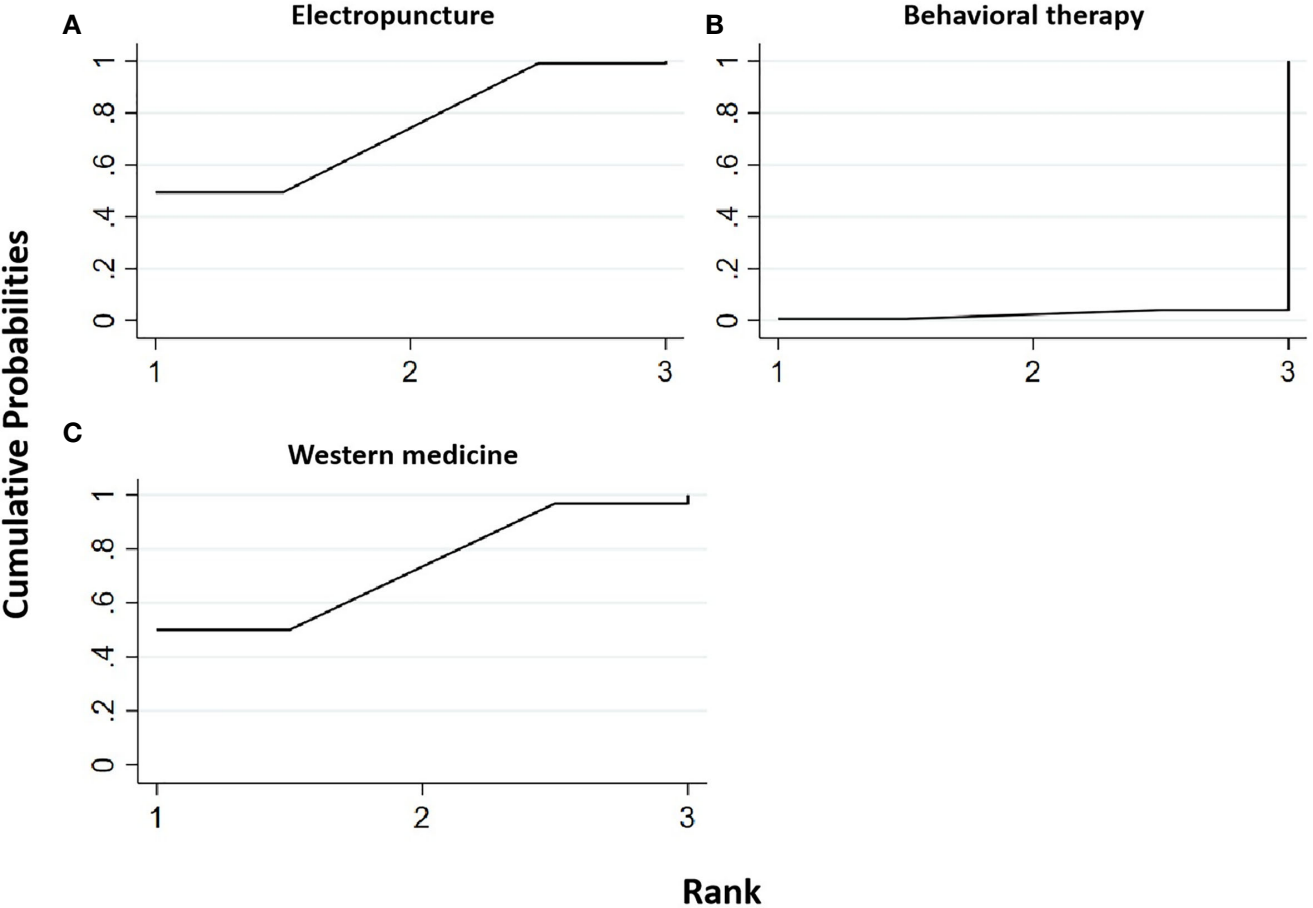
Efficacy ranking and cumulative probability graph of total cholesterol (TC) treated by different types of intervention. Comparison of efficacy ranking and cumulative probability of TC between each kind of intervention. **(A)** Electropuncture, **(B)** behavioral therapy, and **(C)** western medicine.

Two RCTs were included in the statistical analysis of TG, with a total sample of 163 patients. The network relationship is shown in **Figure 2H**. No significant heterogeneity or inconsistency was found in the reticular body of evidence; thus, the consistent fixed-effect model was adopted. The pairwise comparison results and ranking of reticular meta-analysis are shown in **Figure 10** and **Tables 10**, **12**.

**Table 12:** Network meta-analysis ladder table of total cholesterol.

Electropuncture	0.40 (0.06, 0.74)	0.00 (-0.26, 0.26)
**-0.40 (-0.74, -0.06)**	Behavioral therapy	-0.40 (-0.83, 0.03)
-0.00 (-0.26, -.0.26)	0.40 (-0.03, 0.83)	Western medicine

Bold values means the sorting tables represent the area under the curve of the cumulative probability sorting chart, the bold values are the rank of the sorting by different types of intervention in our study. While the number of the ladder table is the relative effect value and 95% CI.

**Table 12** shows that, compared with blank control, electropuncture (MD = -0.40, 95% CI: -0.74, -0.06) caused the decrease in the TC level. No statistical differences were found in the other groups. From **Figure 10** and **Table 10**, we found that electropuncture ranked first in terms of reducing TC in obesity combined with insulin resistance.

#### High-density lipoprotein

**Figure 11:**
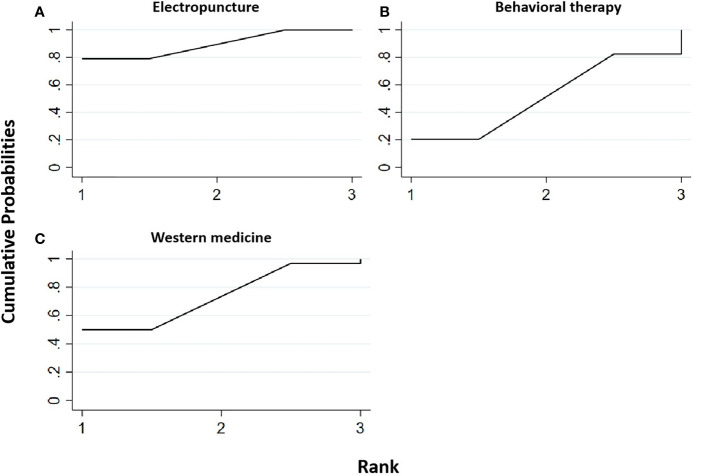
Efficacy ranking and cumulative probability graph of high-density lipoprotein (HDL) treated by different types of intervention. Comparison of efficacy ranking and cumulative probability of HDL between each kind of intervention. **(A)** Electropuncture, **(B)** behavioral therapy, and **(C)** western medicine.

Two RCTs were included in the statistical analysis of HDL, with a total sample of 163 patients. The network relationship is shown in **Figure 2I**. No significant heterogeneity or inconsistency was found in the reticular body of evidence; thus, the consistent fixed-effect model was adopted. The pairwise comparison results and ranking of reticular meta-analysis are shown in **Figure 11** and **Tables 13**, **14**.

**Table 13:** Efficacy ranking of mesh meta-analysis: high-density lipoprotein (HDL) and low-density lipoprotein (LDL).

Treatment	SUCRA	Rank
HDL		
Electropuncture	89.5	1
Behavioral therapy	51.4	2
Western medicine	9.1	3
LDL		
Electropuncture	83.6	1
Western medicine	65.9	2
Behavioral therapy	0.5	3

As shown in the table, the surface under the cumulative ranking curve (SUCRA) represents the area under the curve of the cumulative probability graph of efficacy ranking. The decrease in fasting blood glucose is better, the SUCRA value is bigger, and the efficacy ranking is higher.

**Table 14:** Network meta-analysis ladder table of high-density lipoprotein.

Electropuncture	-0.03 (-0.10, 0.04)	-0.07 (-0.13, -0.01)
0.03 (-0.04, 0.10)	Behavioral therapy	-0.04 (-0.13, 0.05)
**0.07 (0.01, 0.13)**	0.04 (-0.05, 0.13)	Western medicine

Bold values means the sorting tables represent the area under the curve of the cumulative probability sorting chart, the bold values are the rank of the sorting by different types of intervention in our study. While the number of the ladder table is the relative effect value and 95% CI.

**Table 14** shows that, compared with blank control, electropuncture (MD = 0.07, 95% CI: 0.01, 0.13) caused the increase in the HDL level. No statistical differences were found in the other groups. From **Figure 11** and **Table 13**, we found that electropuncture ranked first in terms of increasing HDL in obesity combined with insulin resistance.

#### Low-density lipoprotein

**Figure 12:**
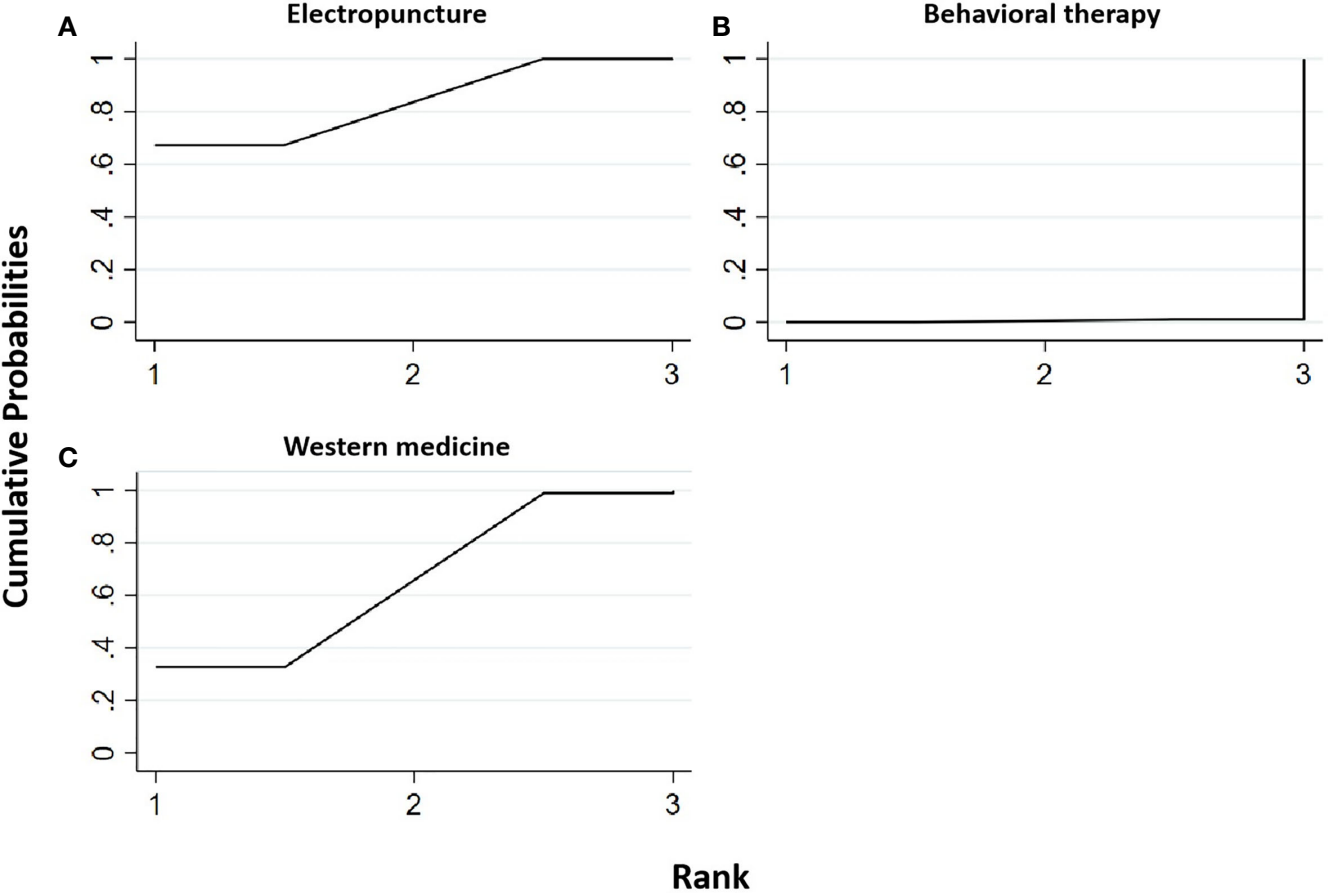
Efficacy ranking and cumulative probability graph of low-density lipoprotein (LDL) treated by different types of intervention. Comparison of efficacy ranking and cumulative probability of LDL between each kind of intervention. **(A)** Electropuncture, **(B)** behavioral therapy, and **(C)** western medicine.

Two RCTs were included in the statistical analysis of LDL, with a total sample of 163 patients. The network relationship is shown in **Figure 2J**. No significant heterogeneity or inconsistency was found in the reticular body of evidence; thus, the consistent fixed-effect model was adopted. The pairwise comparison results and ranking of reticular meta-analysis are shown in **Figure 12** and **Tables 13**, **15**.

**Table 15:** Network meta-analysis ladder table of low-density lipoprotein.

Electropuncture	0.38 (0.15, 0.61)	0.04 (-0.13, 0.21)
**-0.38 (-0.61, -0.15)**	Behavioral therapy	-0.34 (-0.63, -0.05)
-0.04 (-0.21, 0.13)	**0.34 (0.05, 0.63)**	Western medicine

Bold values means the sorting tables represent the area under the curve of the cumulative probability sorting chart, the bold values are the rank of the sorting by different types of intervention in our study. While the number of the ladder table is the relative effect value and 95% CI.

**Figure 13:**
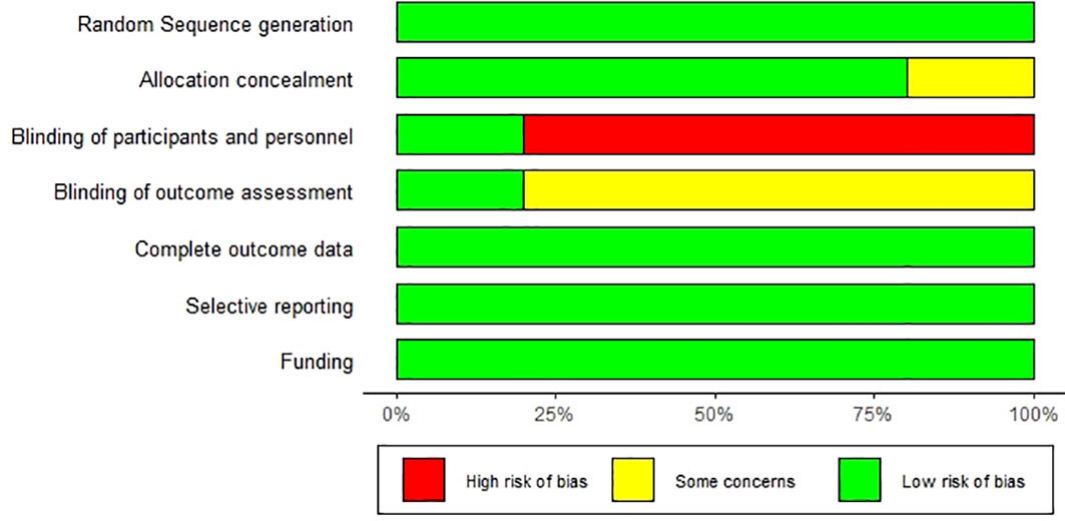
Evaluation for bias risk of the included studies.

**Table 15** shows that, compared with blank control, electropuncture (MD = -0.38, 95% CI: -0.61, -0.15) caused the decrease in the LDL level. However, the curative efficacy of behavioral therapy (MD = 0.34, 95% CI: 0.05, 0.63) was not as good as western medicine. No statistical differences were found in the other groups. From **Figure 13** and **Table 15**, we found that electropuncture ranked first in terms of reducing LDL in obesity combined with insulin resistance.

#### The assessment of literature bias risk

Five RCTs all reported the random sequence generation methods. None of them described distribution concealment, but four RCTs could be considered as having a low risk of bias. In terms of the blind method, four RCTs considered that the subjects did not achieve blinding due to significant differences in intervention measures between the experimental group and the control group. In addition, one RCT clearly described the blind method for the statistical analysis of results, while the other four RCTs did not clearly describe the blind method. Five RCTs had a low risk of bias in the proportion of lost follow-up in each group, selective reporting, and corporate funding. As a whole, except for the blind method, which is difficult to achieve, all the included studies have a low risk of bias. The specific risk of bias is shown in **Figure 13**.

In summary, this study focused on three acupuncture methods in existing studies: electropuncture, acupoint catgut embedding, and acupuncture point patch. The network meta results showed that these three acupuncture methods had significant efficacy in reducing FBG, FINS, HOMA-IR, BMI, waistline, and waist–hip ratio in obesity combined with insulin resistance compared with the blank control group. Meanwhile, compared with the western medicine group, these three acupuncture methods had better efficacy in some outcome indicators. In a series of outcomes related to blood lipid, the efficacy of electropuncture was significantly better than behavioral therapy and western medicine. Except for the fact that acupoint catgut embedding was superior to electropuncture in reducing BMI, there was no statistically significant difference in efficacy among the three acupuncture methods.

## Discussion

Acupuncture, which is among the oldest healing practices in the world, was suggested as an application to a wide range of conditions including musculoskeletal diseases, neurological disorders, gynecological disorders, addictions, and dentistry by the WHO (7). It exerts its effect through the insertion of thin metallic needles at specific points on the body that can be manipulated manually or by electrical stimulation (15).

Electropuncture, consisting of stimulating specific points on the body by inserting thin metal needles into superficial structures with a tiny electrical current, is employed for removing blockages in the flow of vital energy that circulates throughout the body through a system of pathways. Acupoint catgut embedding therapy, involving persistent stimulation produced by a suture with mild irritation in subcutaneous tissue, may be related to a combination of proteolytic enzymes, and macrophage action against the absorbable surgical thread may improve and extend the acupoint stimulation (16). By acupuncture point patch, which is a combination of traditional Chinese medicine, acupuncture, and channels and collaterals in applying Chinese medicine to the corresponding acupoints of the human body, diseases can be prevented and cured.

The current conventional therapeutic strategies for obesity (*i*.*e*., diet, physical exercise, drugs, and bariatric surgery) cannot achieve adequate weight control in all patients. Complementary types of treatment are therefore being tested, and in this context, acupuncture is one of the most rapidly growing complementary therapies. In the USA, the National Institutes of Health consensus panel recommends acupuncture as a useful clinical procedure.

The pathogenesis and pathological process of obesity combined with IR are complex. Currently, it is known that acupuncture can improve obesity combined with IR through multilevel, multisystem, and multitarget synergistic action, but the exact mechanism still needs to be clarified. Due to the influence of acupoint specificity, acupoint compatibility, and acupuncture stimulation parameters on the acupuncture effect and curative effect of obesity combined with insulin resistance (17), the specific internal mechanism is still not comprehensive.

We hereby critically examine major developments in the treatment of obesity combined with IR by acupuncture, which have helped shape our contemporary diagnostic and treatment strategies in obesity combined with IR. Acupuncture, in this study, involved electropuncture, acupoint catgut embedding, and acupuncture point patch, all of which were compared with other acupuncture treatments, drug therapy, or blank control. The results of this study showed that these three acupuncture methods had significant efficacy in reducing FBG, FINS, HOMA-IR, BMI, waistline, and waist–hip ratio in obesity combined with IR compared with the control group. Meanwhile, compared with the western medicine group, these three acupuncture methods had better efficacy in some outcome indicators. In a series of outcomes related to blood lipid, the efficacy of electropuncture was significantly better than behavioral therapy and western medicine, with its respective advantages. According to the above-mentioned research results, different acupuncture methods have obvious advantages in the diagnosis and treatment of obesity combined with IR.

## Conclusion

This study has some limitations: (1) most of the included literatures did not report specific allocation concealment, blind method, and follow-up, which may lead to selection and measurement bias, and (2) the number of RCTs involved in this study was limited, and some literatures did not report safety indicators. Therefore, it is impossible to draw conclusions about the safety of different acupuncture methods. More RCTs with high-quality, multicenter, large-sample randomized controlled trials are needed to explore in depth so as to provide stronger clinical evidence in the future. In addition, (3) at present, only the selection of acupuncture points and treatment modality have more observations on the efficacy. The timing of acupuncture for weight loss treatment is not uniformly reported clinically. Of course, the existence of different treatment durations will affect the final evaluation of efficacy. More quality literature on the duration of acupuncture treatment for obesity is expected to follow.

In summary, our study evaluated the clinical efficacy of different acupuncture methods commonly used in clinical treatment on obesity combined with IR. Different acupuncture methods have good advantages in both overall efficacy score and single index evaluation, providing strong objective evidence for obesity combined with IR in the future.

## Data availability statement

The raw data supporting the conclusions of this article will be made available by the authors, without undue reservation.

## Author contributions

CX, JL, and YC conceived and designed the experiments. JC, YG, LY, MH, NL, and YL analyzed and interpreted the data. MH, NL, and YL revised the data analysis and interpretation. JC, YG, and LY wrote the article. All authors contributed to the article and approved the submitted version.

## Funding

This work was supported by grants from State Key Laboratory of Dampness Syndrome of Chinese Medicine Special Fund (SZ2021ZZ08), Guangdong Provincial Bureau of Chinese Medicine (20225020), the Fund of Guangzhou University of Chinese Medicine (2021YJZX012, 2022YBA06), the Guangdong Provincial Hospital of Chinese Medicine Fund (YN2019ZWB01, YN2020MS02, YN2019ML01, BAQZJYJZX [2019] 007) and Research Fund for Zhaoyang Talents of Guangdong Provincial Hospital of Chinese Medicine (ZY2022KY10, ZY2022YL04).

## Conflict of interest

The authors declare that the research was conducted in the absence of any commercial or financial relationships that could be construed as a potential conflict of interest

## Publisher’s note

All claims expressed in this article are solely those of the authors and do not necessarily represent those of their affiliated organizations, or those of the publisher, the editors and the reviewers. Any product that may be evaluated in this article, or claim that may be made by its manufacturer, is not guaranteed or endorsed by the publisher.
