# Supplementary material for: Network meta-analysis of curative efficacy of different acupuncture methods on obesity combined with insulin resistance
Source: Front Endocrinol (Lausanne). 2022 Sep 2;13:968481. doi: 10.3389/fendo.2022.968481 (PMC9481269; doi:10.3389/fendo.2022.968481)
Supplement: Supplementary file 1 [file Table_1.docx]

| **Supplementary Table S1 Outcome report of included studies.** | **LDL** | √ | √ |  |  | √ | "√" in the figure represents the outcome index corresponding to the column in the corresponding included study report. The grid marked with blue shadow indicates that the data can construct mesh evidence body or indirect comparative evidence body, which can be included in the analysis. |
| --- | --- | --- | --- | --- | --- | --- | --- |
|  | **HDL** | √ | √ |  |  | √ |  |
|  | **TC** | √ | √ |  |  | √ |  |
|  | **TG** | √ | √ |  |  | √ |  |
|  | **Waist-hip ratio** |  |  | √ | √ |  |  |
|  | **Waistline** |  |  | √ | √ | √ |  |
|  | **Weight** |  |  | √ |  | √ |  |
|  | **BMI** |  |  | √ | √ | √ |  |
|  | **HOMA-IR** | √ | √ | √ | √ | √ |  |
|  | **FINS** | √ | √ | √ | √ | √ |  |
|  | **FBG** | √ | √ | √ | √ | √ |  |
|  | **Treatment** | electropuncture  VS  behavioral therapy | electropuncture  VS  western medicine | electropuncture  VS  the blank control | acupoint catgut embedding  VS  the blank control | acupoint catgut embedding  VS  acupuncture point patch |  |
|  | **Study** | Li2018 | Li2019 | Ni2022 | Wan2022 | Zhou2020 |  |
